# GFI1 regulates hair cell differentiation by acting as an off-DNA transcriptional co-activator of ATOH1, and a DNA-binding repressor

**DOI:** 10.1038/s41598-022-11931-0

**Published:** 2022-05-12

**Authors:** Hsin-I Jen, Sunita Singh, Litao Tao, Helen R. Maunsell, Neil Segil, Andrew K. Groves

**Affiliations:** 1 Department of Neuroscience, Baylor College of Medicine, 1 Baylor Plaza, Houston, TX 77030 USA; 2 Department of Molecular and Human Genetics, Baylor College of Medicine, BCM295, 1 Baylor Plaza, Houston, TX 77030 USA; 3 Program in Development, Disease Models & Therapeutics, Baylor College of Medicine, 1 Baylor Plaza, Houston, TX 77030 USA; 4 Department of Stem Cell Biology and Regenerative Medicine, Keck School of Medicine of the University of Southern California, Eli and Edythe Broad Center for Regenerative Medicine and Stem Cell Biology at USC, 1425 San Pablo St., Los Angeles, CA 90033 USA; 5 Caruso Department of Otolaryngology – Head and Neck Surgery, Keck School of Medicine of the University of Southern California, 1450 San Pablo St., Suite 5100, Los Angeles, CA 90033 USA; 6 Present Address: Department of Biomedical Sciences, Creighton University School of Medicine, Omaha, NE USA

**Keywords:** Developmental biology, Molecular biology

## Abstract

GFI1 is a zinc finger transcription factor that is necessary for the differentiation and survival of hair cells in the cochlea. Deletion of *Gfi1* in mice significantly reduces the expression of hundreds of hair cell genes: this is a surprising result, as GFI1 normally acts as a transcriptional repressor by recruiting histone demethylases and methyltransferases to its targets. To understand the mechanisms by which GFI1 promotes hair cell differentiation, we used CUT&RUN to identify the direct targets of GFI1 and ATOH1 in hair cells. We found that GFI1 regulates hair cell differentiation in two distinct ways—first, GFI1 and ATOH1 can bind to the same regulatory elements in hair cell genes, but while ATOH1 directly binds its target DNA motifs in many of these regions, GFI1 does not. Instead, it appears to enhance ATOH1’s transcriptional activity by acting as part of a complex in which it does not directly bind DNA. Second, GFI1 can act in its more typical role as a direct, DNA-binding transcriptional repressor in hair cells; here it represses non-hair cell genes, including many neuronal genes. Together, our results illuminate the function of GFI1 in hair cell development and hair cell reprogramming strategies.

## Introduction

Mechanosensory hair cells of the inner ear are secondary sensory receptor cells—they contain specializations to detect force and develop graded receptor membrane potentials in response to mechanical stimuli. They form synapses with sensory neurons and release synaptic vesicles in response to calcium influx from voltage-gated calcium channels^1,2^. However, they do not form axons or dendrites and do not fire action potentials. This distinguishes them—and vertebrate photoreceptors, which are also secondary receptor cells—from their invertebrate counterparts^3^. As vertebrates diverged from invertebrates, evidence suggests that secondary receptor cells preserved some elements of neuronal gene regulatory networks, but modified them to allow the emergence of new cell types^4,5^.

The *Drosophila atonal* gene acts as a proneural transcription factor to specify mechanosensory chordotonal organs (including the mechanosensory neurons themselves) as well as photoreceptors^6–8^. The vertebrate homologue of *atonal*, *Atoh1*, is necessary and sufficient for the differentiation of hair cells from prosensory progenitors^9–16^. Other members of the *Drosophila* mechanosensory gene regulatory network are conserved in vertebrates, such as the zinc finger transcription factor *senseless*, and its vertebrate homologues *Gfi1* and *Gfi1b*^17–19^. *Drosophila* Senseless interacts with Atonal through its Zn finger motifs, and enhances the ability of Atonal to activate transcription of proneural target genes^20–23^. It can also bind directly to DNA through its zinc fingers, and acts as a transcriptional repressor, although its repressive role in proneural differentiation appears to be required mainly in cells expressing low levels of Senseless^20^. In contrast, vertebrate GFI1 proteins have acquired a SNAG repression domain at their N-terminus and their primary role appears to act as repressors by recruiting LSD1/CoREST histone demethylases and HDAC histone deacetylases^24–29^. Indeed, recent data from *Gfi1* knockout mice suggest that one function of *Gfi1* in inner ear hair cells is to repress neuronal programs of differentiation^30^. This raises the question of whether *Gfi1’*s function in hair cell gene regulatory networks is simply to repress some aspects of neuronal differentiation, with other aspect of the neuronal phenotype, such as the ability to form synapses, remaining extant in hair cells. Alternatively, it is possible that GFI1 has retained the ability of Senseless to act as a co-activator of ATOH1 and positively promote the expression of hair cell genes. In either case, it is not clear if GFI1 regulates hair cell differentiation by directly engaging DNA target sequences and/or acting as part of an activation complex with ATOH1 without directly binding DNA. These questions have acquired new salience with the advent of technologies to reprogram cells to a hair cell fate. For example, combined expression of *Atoh1, Gfi1* and *Pou4f3* is sufficient to convert ES cells or fibroblasts into hair cell-like cells^31–34^ and also to reprogram cells of the inner ear to a hair cell-like fate^35^, and the addition of a fourth transcription factor, Six1, to this cocktail can more efficiently reprogram embryonic and postnatal fibroblasts into hair cell-like cells^36^. Finally, adenoviral delivery of *Atoh1* and *Gfi1* to the deafened inner ear has recently been shown to promote the formation of new hair cell-like cells in the adult mouse cochlea at a significantly higher efficiency than *Atoh1* alone^37^.

To address the question of the roles and mechanism of *Gfi1* in hair cell differentiation, we have examined genes positively and negatively regulated by *Gfi1* in *Gfi1* null mice^30^ and analyzed its binding targets in developing hair cells using CUT&RUN. We show that GFI1 can act as a transcriptional repressor by binding DNA directly, but that it is also necessary to positively regulate hair cell gene transcription. It appears to do so as part of a complex with ATOH1 and E box binding proteins such as E47, although our data suggests GFI1 does not directly bind to ATOH1 in this complex. Our data reveal new insights into the mechanism of GFI1 action in hair cells and provide insights into its potential use in inducing hair cell identity as part of therapeutic reprogramming strategies.

## Results

### GFI1 can act as a positive and negative regulator of hair cell gene expression

**Figure 1:**
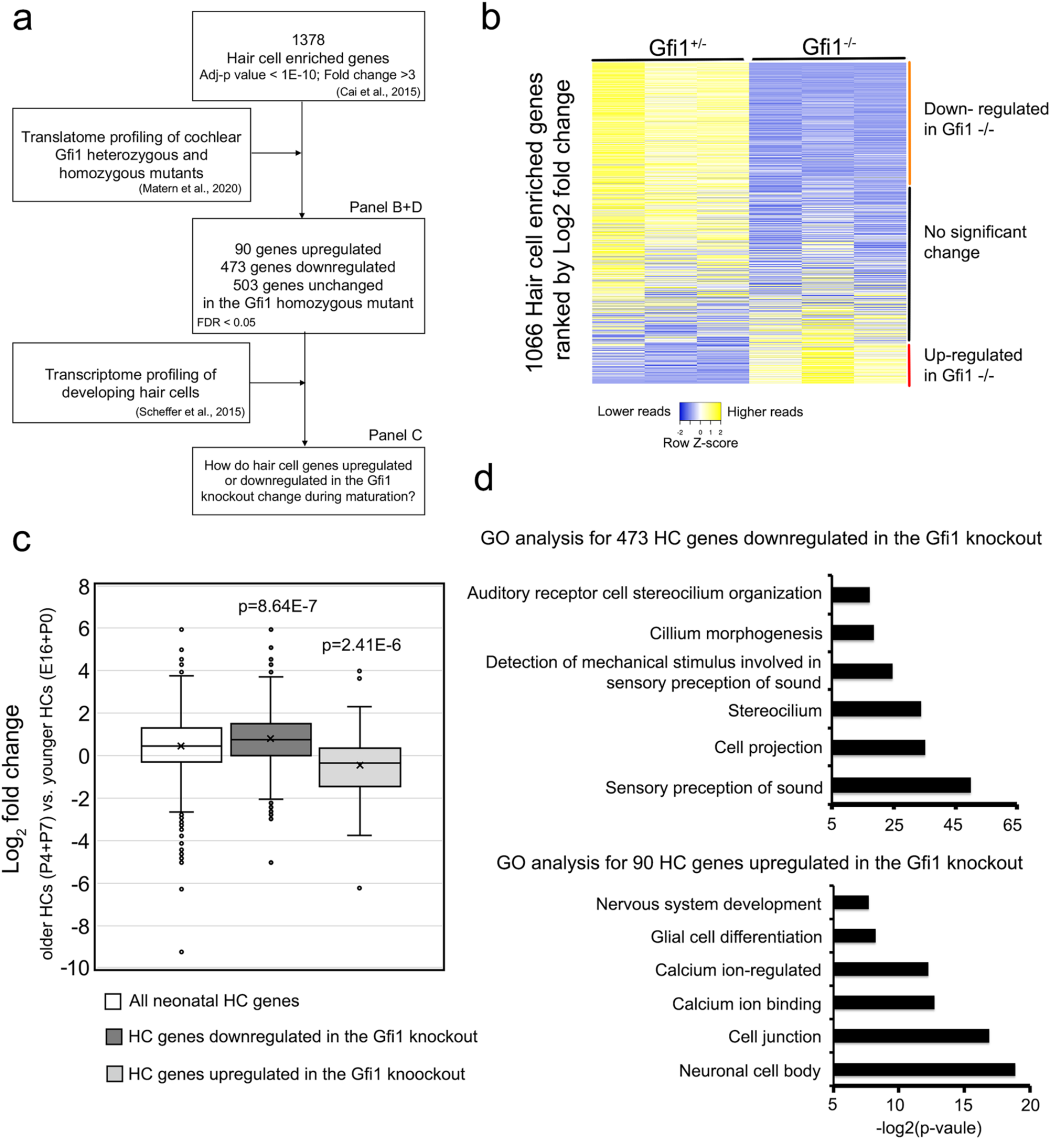
GFI1 differentially regulates expression of hair cell genes during inner ear development. (**a**) Overview of the analysis workflow: We used our previously published RNA-seq data from neonatal hair cells^38^ to identify a list of 1378 genes enriched in hair cells. We then cross-referenced this list with RNA-seq translatome data from *Gfi1* mutant or heterozygote hair cells^30^ to identify genes that were up-or-down-regulated in *Gfi1* null mice. We then examined how expression of these GFI1-regulated genes varied in hair cells with developmental time, but using data from E16, P1, P4 and P7 purified hair cells^40^. (**b**) Heat map for the expression of hair cell-enriched genes in *Gfi1* heterozygous and null mice showing. The orange-labeled cluster represents genes significantly downregulated in *Gfi1* homozygous mutant hair cells, the red-labeled cluster represents genes that are significantly upregulated in the *Gfi1* mutant, and the black-labeled cluster shows genes that are not significantly changed in mutant hair cells. (**c**) Box plot showing that the 473 hair cell genes that are down-regulated in the *Gfi1* knockout tend to be expressed at higher levels in more mature (P4 and P7) hair cells, while the 90 hair cell genes that are up-regulated in the *Gfi1* knockout tend to be expressed at a lower level in more mature hair cells. Statistical significance was assessed by a Student t-test, comparing the Log_2_ fold change (FC) values of each gene group to the log_2_ FC values of all neonatal hair cell genes. (**d**) Gene ontology analysis of hair cell-enriched genes that are either down-regulated (473 genes) or up-regulated (90 genes) in the *Gfi1* knockout.

To understand how GFI1 regulates hair cell gene expression, we used our previously published RNA-seq data from purified neonatal cochlear hair cells^38^ to identify 1378 genes enriched in hair cells compared to other cell types in the cochlea (adjusted p-value < E−10; fold change > 3; Supplementary Table 1 Sheet 1). We used published RNA-seq data from neonatal *Gfi1* knockout mouse hair cells^30^ to ask how the expression of cochlear hair cell genes changed in the *Gfi1* knockout (Fig. 1a). We found 473 hair cell genes were downregulated in the *Gfi1* knockout cochlea, while 90 hair cell genes were up-regulated (false discovery rate < 0.05; Fig. 1b; Supplementary Table 1, Sheet 2). During normal hair cell development, some early hair cell genes, including *Atoh1*, are down-regulated in differentiating hair cells shortly after birth, while other genes continue to be expressed at high levels in mature hair cells^15,38,39^. To investigate if GFI1 differentially regulates hair cell genes based on whether they are transiently or stably expressed in hair cells, we obtained the expression level of these genes in hair cells at four different developmental stages (E16.5, P0, P4 and P7) from the SHIELD database of hair cell gene expression (Fig. 1a; http://shield.hms.harvard.edu;^40^. We found that the 90 hair cell genes that were negatively regulated by GFI1 (i.e., up-regulated in the *Gfi1* knockout) tended to have lower expression in more mature (P4 and P7) hair cells, while the 473 hair cell genes that were positively regulated by GFI1 (i.e., down-regulated in the *Gfi1* knockout) tended to be expressed at higher levels in more mature hair cells (Fig. 1c).

We next performed gene ontology analysis to understand what types of genes were enriched in the 473 hair genes positively regulated by GFI1. These genes were enriched for GO terms associated with the sensory perception of sound (GO:0007605; p = 9.0E−16), stereocilia (GO:0032420; p = 6.6E−11), and detection of mechanical stimulus involved in sensory perception of sound (GO:0050910; p = 3.9E−8; Fig. 1d). These results suggested that GFI1 directly or indirectly positively regulates genes involved in hair cell maturation. In contrast, gene ontology analysis for the 90 genes that were down-regulated in the *Gfi1* knockout showed enrichment for GO terms associated with neuronal cell body (GO:0043025; p = 2.09E−0.6), synapse (GO:0045202; p = 4.6E−5), and calcium ion-regulated exocytosis of neurotransmitter (GO:0048791; p = 2.08E−4) (Fig. 1d). In addition to hair cell genes, more than 672 non-hair cell genes were also upregulated in the *Gfi1* knockout cochlea (FDR < 0.05). These genes were enriched for GO terms associated with nervous system development (GO:0007399; p = 5.88E−11), axon guidance (GO:0007411; p = 1.03E−7), and dendrite morphogenesis (GO:0048813; p = 4.10E−7; Supplementary Fig. 1). Similar to previous findings^30^, this analysis showed that GFI1 functions to attenuate the expression of many neuronal genes in neonatal hair cells. Together, our results suggest that GFI1 can act as both a positive and negative regulator in hair cells to orchestrate the correct expression and timing of hair cell genes in the developing cochlea, and that its repressive function in hair cells may serve to attenuate the expression of developmental or neuronal genes as hair cells mature.

### GFI1 acts as a direct regulator of hair cell genes by binding at hair cell gene loci

**Figure 2:**
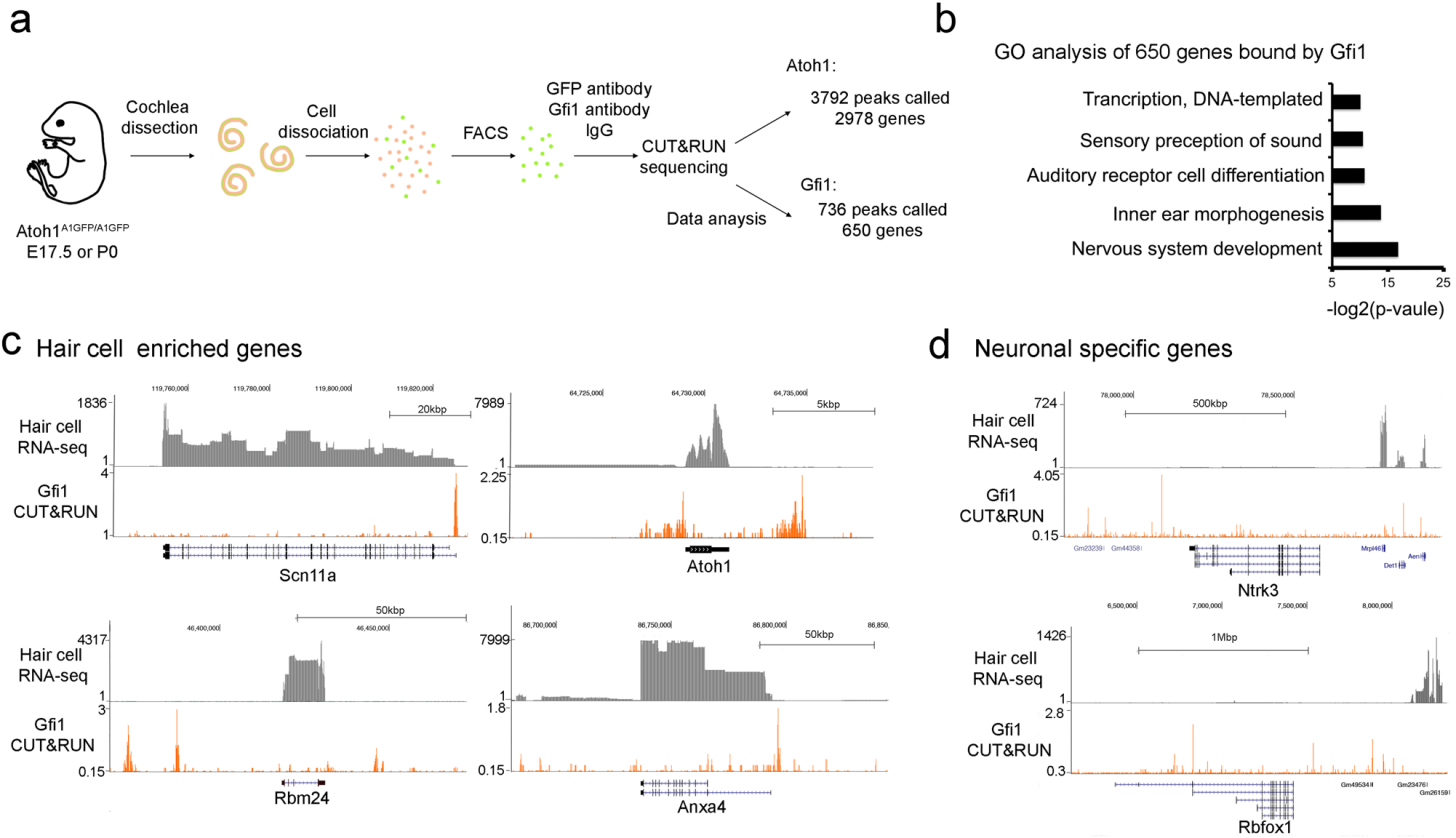
GFI1 directly binds to many genomic loci that it positively or negatively regulates. (**a**) Diagram of the CUT&RUN sequencing experiment. Cochleas from *Atoh1*^*A1GFP*^ mice in which the hair cell transcription factor ATOH1 is fused to GFP were dissected, dissociated into single cells and FACS-sorted. Purified hair cells were processed for CUT&RUN sequencing with antibodies to either GFP (to identify ATOH1 targets), GFI1, or an IgG control. (**b**) Gene ontology analysis of the 650 GFI1 binding genes. (**c**) Genomic browser track of CUT&RUN sequencing showing examples of GFI1 binding to loci near four hair cell genes: *Scn11a, Atoh1, Rbm24,* and *Anxa4*. With the exception of *Atoh1*, they are all downregulated in the Gfi1 knockout cochlea^30^. (**d**) Genomic browser track of CUT&RUN sequencing showing examples of GFI1 binding loci near known neuronal-specific genes, Ntrk3 and Rbfox that are upregulated in the *Gfi1* knockout cochlea^30^. As shown in the RNA-seq traces, these two genes are normally not expressed in the neonatal hair cells, so the trace scale is expanded in both cases to show RNA-seq traces from nearby genes as a positive control.

Our analysis of *Gfi1* knockout mice identified hair cell genes that are positively and negatively regulated by GFI1, but did not distinguish which genes are *directly* regulated by GFI1, as opposed to being downstream of *Gfi1* in the hair cell gene regulatory network. To identify sites in hair cell chromatin that were occupied by GFI1 protein, we purified hair cells from late embryonic or neonatal mouse cochleas using *Atoh1-GFP* fusion reporter mice^41^ and performed CUT&RUN^42^ to identify DNA regions to which GFI1 was bound using antibodies to Gfi1 or IgG control. After normalizing to the IgG control, we called a total of 736 GFI1 peaks using MACS2 (Supplementary Table S2 Sheet 1). Annotation of these peaks to nearby genes identified 650 genes associated with these GFI1 binding sites (Supplementary Table S2 Sheet 1). Gene ontology analysis of this gene collection revealed enrichment for GO terms associated with nervous system development (GO:0007399; p = 8.30E−06), inner ear morphogenesis (GO:0042472; p = 6.82E−05) and auditory receptor cell differentiation (GO:0042491; p = 5.30E−04; Fig. 2b). Examples of loci where GFI1 is bound in hair cells are shown in Fig. 2c. Examples of neuronal genes that are bound directly and repressed by GFI1 are shown in Fig. 2d.

**Figure 3:**
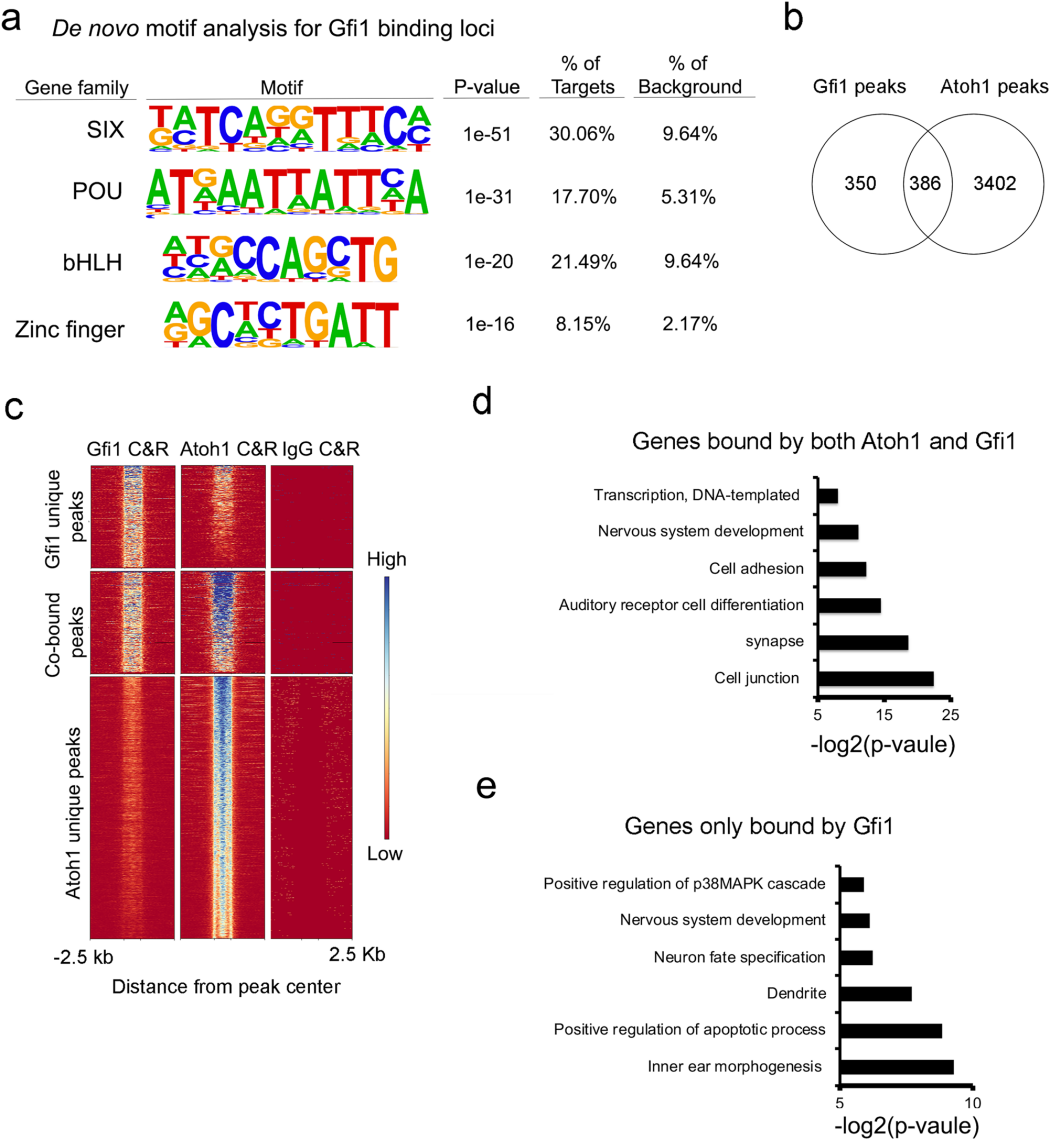
GFI1 and ATOH1 bind to many common genomic sites in hair cells. (**a**) De novo motif analysis of 732 GFI1 binding loci. Of note, the most significantly enriched motifs in the GFI1-bound peaks are for SIX, POU and bHLH protein families, with zinc finger family protein motifs (of which GFI1 is a member) are only found in 8.15% of targets. (**b**) Venn diagram of the peaks obtained in CUT&RUN experiments for GFI1 and ATOH1 binding, showing 386 overlapping loci that are bound by both GFI1 and ATOH1. (**c**) Heat map showing individual called peaks from GFI1 and ATOH1 CUT&RUN analysis shown in B. (**d**) Gene ontology analysis of and the 341 ATOH1 and GFI1 co-bind genes. (**e**) Gene ontology analysis of the 309 GFI1 uniquely binding genes.

To determine which hair cell genes are either directly repressed or activated by GFI1 during hair cell development, we compared our GFI1 CUT&RUN data with the hair cell genes that were up-or down-regulated in *Gfi1* knockout mice. We found GFI1 bound to 51 hair cell genes that were downregulated in the *Gfi1* knockout (i.e., positively regulated by GFI1) and 8 hair cell-enriched genes that were upregulated in the *Gfi1* knockout (i.e., negatively regulated by GFI1; Supplementary Table S3). These results suggest that GFI1 can function to both directly activate and directly repress hair cell genes. To identify which transcription factors GFI1 might be co-operating with in hair cells, we performed a de novo* motif* analysis on the 736 GFI1 peaks from our CUT&RUN analysis. A number of motifs for families of transcription factors associated with hair cell development were enriched in the peaks, including motifs for SIX family proteins (p-value = 1E−51), POU family proteins (p-value = 1E−31) and bHLH family proteins (p-value = 1E−20; Fig. 3a). Although motifs for zinc-finger transcription factor family proteins (of which GFI1 is a member) were enriched in the peaks, their enrichment was significantly lower than for the other transcription factor families described above (p-value = 1e−16).

### GFI1 positively regulates hair cells genes by binding with ATOH1 at the same genomic loci

In *Drosophila*, the GFI1 homolog *Senseless* acts as a positive co-regulator of gene expression in concert with proneural transcriptional factors such as the *Atoh1* homolog *atonal*^20^. However, it has not been established whether mammalian GFI1 and ATOH1 interact together in cochlear hair cell development. When we performed a known motif analysis for bHLH transcription factor sites on peaks that were bound by GFI1, we found that the “AtEAM” sequence motif associated with ATOH1^43^ is enriched in the GFI1-bound peaks (p-value = 1E−11; Supplementary Fig. 2). To confirm if GFI1 interacts with ATOH1 in vivo, we first examined whether they can be localized to similar genomic sites in hair cells. We identified direct targets of ATOH1 in purified hair cells using CUT&RUN (Figs. 2a, 3c). We identified 3792 ATOH1-bound peaks using MACS2 (Fig. 3b, Supplementary Table S2 Sheet 2). 386 out of the 736 GFI1-bound peaks overlapped with ATOH1 bound peaks (Fig. 3b,c, Supplementary Table 4). These results suggested that GFI1 and ATOH1 interact together at some genomic loci in hair cells.

We performed gene ontology analysis on the 341 genes that were associated with the loci bound by both ATOH1 and GFI1. Some of the top hits included gene sets that were enriched in auditory receptor cell differentiation (GO:0042491; p = 4.40E−5), cell adhesion (GO:0007155; p = 2.01E−4), and nervous system development (GO:0007399; p = 4.18E−4) (Fig. 3D). In contrast, the gene ontology analysis for the 309 genes that are associated with the loci only bound by GFI1 included genes sets for positive regulation of apoptotic process (GO:0043065; p = 0.0022) and positive regulation of p38MAPK cascade (GO:1900745; p = 0.0167) (Fig. 3E). These results showed that loci where GFI1 and ATOH1 bind together are associated with genes expressed during hair cell differentiation, confirming the idea that GFI1 can promote hair cell differentiation by cooperating with ATOH1.

**Figure 4:**
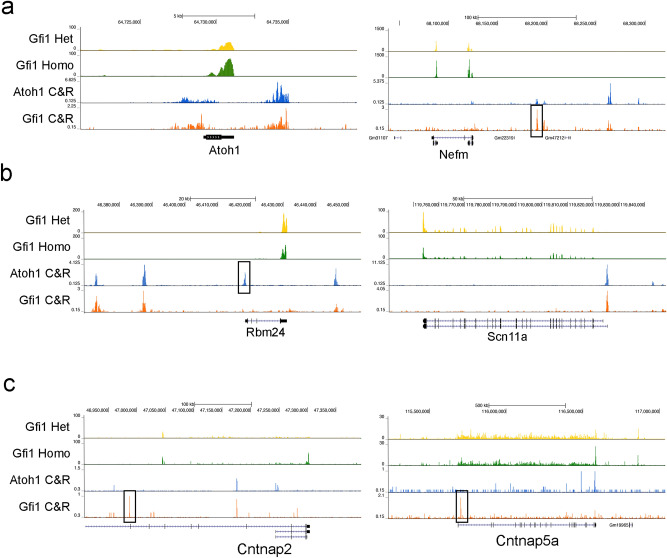
Examples of hair cell and non-hair cell enriched genes that are bound and regulated by GFI1 and ATOH1. (**a**) Genomic browser tracks of RNA-seq and CUT&RUN sequencing of two hair cell genes containing both ATOH1 and GFI1 binding sites that are up-regulated in the *Gfi1* knockout: *Atoh1* (Log_2_ fold change in homozygous (homo) vs. heterozygous (het) *Gfi1* mutants (LFC) = 0.42; False discovery rate (FDR) = 0.0492) and *Nefm* (LFC = 0.84; FDR = 0.000127). Box shows a peak that is uniquely bound by GFI1. (**b**) Genomic browser tracks of RNA-seq and CUT&RUN sequencing of two hair cell genes containing both ATOH1 and GFI1 binding sites that are down-regulated in the *Gfi1* knockout: *Rbm24* (LFC = − 0.85; FDR = 5.25E−07) and *Scn11a* (LFC = − 0.5; FDR = 0.00245). Box shows a peak that is uniquely bound by ATOH1. (**c**) Genomic browser tracks of RNA-seq and CUT&RUN sequencing of neuronal (non-hair cell) genes containing both ATOH1 and GFI1 binding sites that are upregulated in the *Gfi1* knockout: *Cntnap2* (LFC = 2.59; FDR = 3.79E−32) and *Cntnap5a* (LFC = 0.79; FDR = 6.23E−05). Boxes show peaks that are uniquely bound by Gfi1.

To understand how GFI1 positively regulates these ATOH1 and GFI1 co-bound hair cell-enriched genes, we cross-referenced our CUT&RUN data with previous published *Gfi1* knockout data^30^. We found that ATOH1 and GFI1 both bind to 44 out of 1378 hair cell genes. Interestingly, only 4 of these genes are upregulated in the *Gfi1* knockout (*Insm2, Nefm, Camta1, Atoh1*, Fig. 4a), whereas the other 40 genes were downregulated in the *Gfi1* knockout (examples shown in Fig. 4b). This result further suggests that GFI1 mostly functions to enhance expression of hair cell genes when cooperating with ATOH1. We also found GFI1 directly binding to 39 non-hair-cell genes that were upregulated in the Gfi1 knockout (examples shown in Fig. 4c).

**Figure 5:**
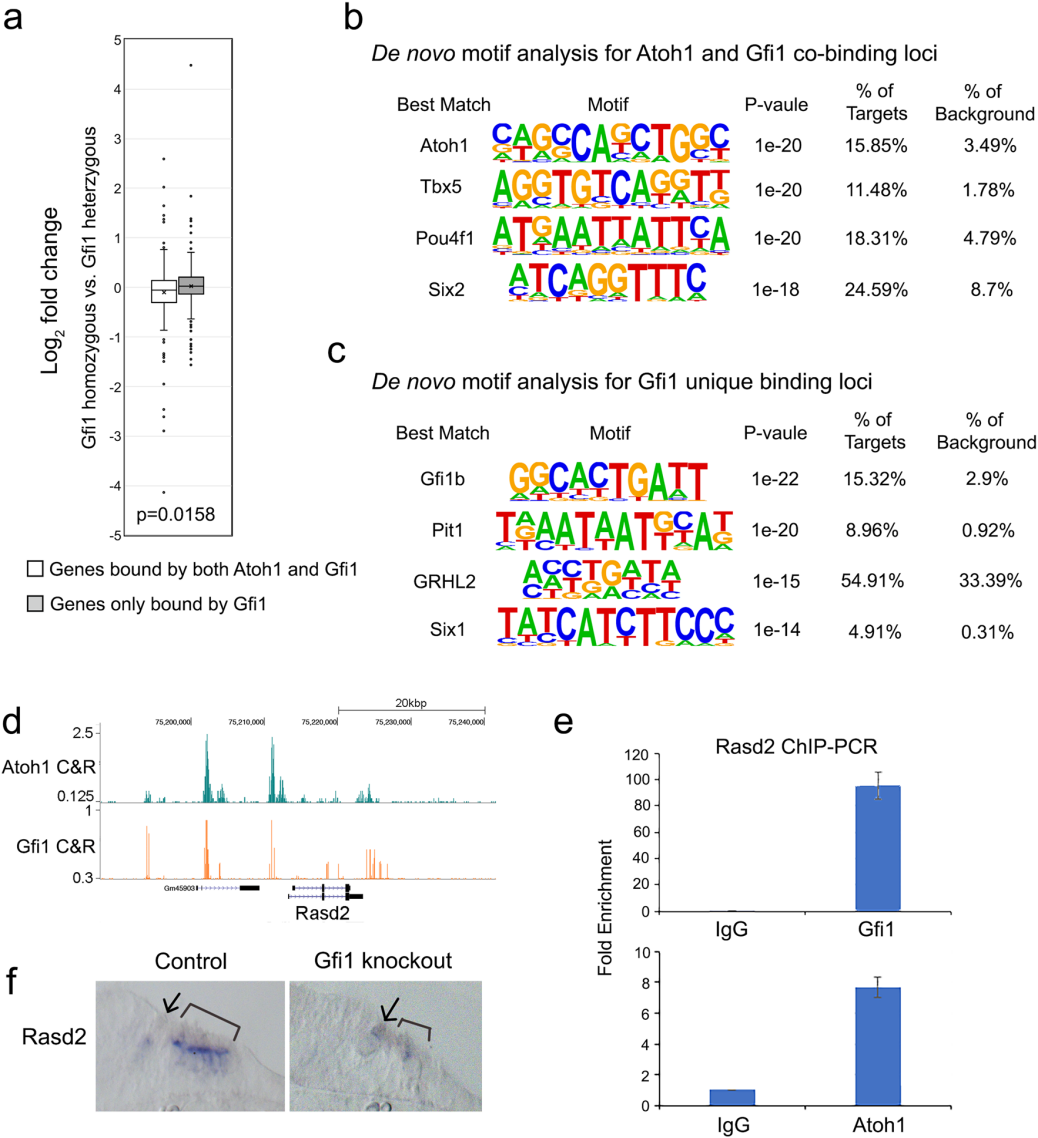
GFI1 positively regulate many hair cell genes without evidence for direct binding to DNA. (**a**) Box plot showing that genes bound by both ATOH1 and GFI1 are more likely to be down-regulated in Gfi1 mutant hair cells (i.e. positively regulated by GFI1), while genes bound by only GFI1 are more likely to be up-regulated in Gfi1 mutant hair cells (i.e. negatively regulated by GFI1). Statistical significance was assessed by a Student t-test, comparing the log_2_ fold change (FC) values. (**b**) De novo motif analysis of the 386 loci bound by both ATOH1 and GFI1. Although ATOH1 DNA recognition motifs are highly enriched in these peaks, Gfi1 DNA recognition motifs are not. (**c**) De novo motif analysis of the 350 loci bound by only by GFI1. Here, the most highly enriched motif is a GFI1b recognition motif. (**d**) Genomic browser track of CUT&RUN sequencing showing both ATOH1 and GFI1 bind to loci near the hair cell gene *Rasd2*. (**e**) ChIP-QPCR analysis of ATOH1 and GFI1 binding to the same distal regulatory elements in the *Rasd2* gene. Cochlear epithelium from neonatal *Atoh1-GFP* mice were processed for ChIP using antibodies for GFP (to pull down ATOH1-bound DNA) and GFI1, with IgG as a negative control. (**f**) In situ hybridization of Rasd2 mRNA in P0 *Gfi1* wild type and mutant cochlea. Arrow marks inner hair cells, brackets mark the outer hair cell region.

To investigate if GFI1 differentially regulates gene expression based on whether it interacts with ATOH1, we compared how the expression level of the genes that were bound by ATOH1 and GFI1 versus those bound by GFI1 alone were altered in *Gfi1* knockout cochleas. We found that the 238 genes that were bound by ATOH1 and GFI1 were preferentially downregulated in the *Gfi1* knockout cochlea, while the 223 genes were bound by only GFI1 tended to be expressed at higher levels in the *Gfi1* knockout cochlea (Fig. 5a). These suggest that GFI1 tends to repress genes when acting alone and tends to activate genes when acting with ATOH1. We next performed de novo motif analysis for the gene loci that were bound by ATOH1 and GFI1 versus those bound by GFI1 alone. The top 3 motifs enriched in the gene loci that were bound by both ATOH1 and GFI1, included ATOH1 (p-value = 1E−20; 15.85% of targets), Tbx5 (p-value = 1E−20; 11.48% of targets) and Pou4f1 (p-value = 1E−20; 18.31% of targets; Fig. 5b). Of note, GFI1 binding motifs were not enriched in this analysis. In contrast, the top 3 motifs that were bound by GFI1 but not ATOH1 included Gfi1b (p-value = 1E−22; 15.31% of targets), Pit1 (p-value = 1E−20; 8.96% of targets) and GRHL2 (p-value = 1E−15; 54.91% of targets) (Fig. 5c). These results suggest GFI1 may interact with ATOH1 without binding directly to DNA, but that it binds directly to DNA when regulating gene expression independently of ATOH1. This form of “off-DNA” interaction also been observed between the *Drosophila* homologues of ATOH1 and GFI1, Atonal and Senseless^20^.

As an illustration of this “off-DNA” function of GFI1, we examined the hair cell gene, *Rasd2*, known to be a direct target of ATOH1^38,44^. A previous study showed that *Rasd2* is downregulated in the *Atoh1* knockout cochlea^38^. The trace from our CUT&RUN data showed GFI1 binds to *Rasd2* at the same distal regulatory regions where ATOH1 binds (Fig. 5d). We confirmed these results with ChIP-QPCR on dissected cochlear epithelium from *Atoh1-GFP* fusion mice using antibodies to GFI1 and to GFP to immunoprecipitate DNA bound by GFI1 or ATOH1-GFP (Fig. 5e). We performed an independent motif screen with known ATOH1 and GFI1 motifs on the four peaks that are bound by both transcription factors. We were only able to identify the ATOH1 binding motifs but not GFI1 binding motifs in the four peaks (Supplementary Fig. 3), suggesting that GFI1 may act as an ATOH1 co-activator to regulate Rasd2 without directly binding to DNA. To test whether *Gfi1* is necessary for the transcription of *Rasd2* in addition to *Atoh1*, we performed in situ hybridization of *Rasd2* in the *Gfi1* knockout cochlea. *Rasd2* is downregulated in the *Gfi1* knockout cochlea compared to controls (Fig. 5f). These results suggested that ATOH1 and GFI1 directly regulate *Rasd2*, but whereas ATOH1 does so by binding DNA directly, GFI1 does not, and that both transcription factors are necessary for its expression in hair cells.

### GFI1 acts as an ATOH1 co-activator without directly binding DNA or ATOH1

**Figure 6:**
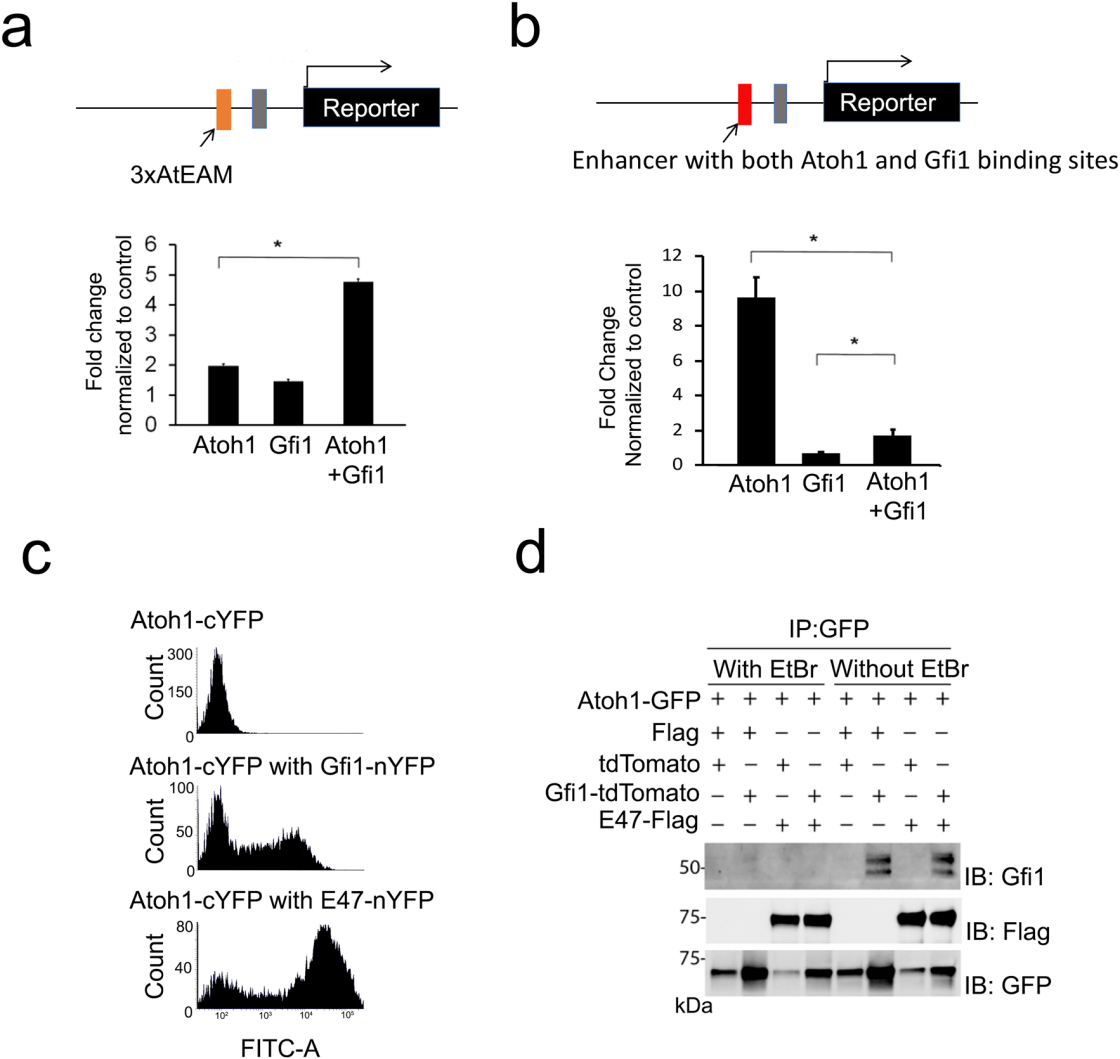
GFI1 enhances the transcription activity of ATOH1 in vitro in an off-DNA mechanism. (**a**) Luciferase assay using a reporter plasmid pNl3.1-3xAtEAM containing an artificial enhancer with 3xAtEAM domains (orange box) in front of a minimal promoter (gray box). The addition of GFI1 significantly enhances the transcriptional activation of the reporter construct by ATOH1, despite the fact that the enhancer contains no Gfi1 binding sites. Statistical significance was assessed by a Student t-test, comparing the average fold change after normalized to the control group each time. (*p < 0.01). (**b**) Luciferase assay using a reporter plasmid pNl3.1-AGmotifs containing an artificial enhancer with both ATOH1 and Gfi1 binding motifs (red box) in front of a minimal promoter (gray box). In this case, addition of GFI1 significantly attenuates the transcriptional activity of ATOH1. Statistical significance was assessed by a Student t-test, comparing the average fold change after normalized to the control group each time. (*p < 0.01). (**c**) Bimolecular fluorescence complementation assay of DAOY cells transfected with Atoh1-cYFP (top), Atoh1-cYFP and GFI1-nYFP (middle) and Atoh1-cYFP and E47-nYFP (bottom). Atoh1-cYFP protein can bind to E47-nYFP, giving a significantly enhanced fluorescence. However, the interaction between Atoh1-cYFP and GFI1-nYFP is significantly weaker. (**d**) Co-immunoprecipitation of ATOH1, GFI1 and E47 in 293 T cells. pCDNA3.1-Atoh1-GFP was transfected into 293 T cells with pEF-Gfi1-tdTomato or pCDNA3.1-E47-Flag. The ATOH1-GFP fusion protein was immunoprecipitated with GFP antibodies, and the blot probed with antibodies to GFI1, FLAG or GFP. GFI1 could be immunoprecipitated with ATOH1 in the presence of non-specific DNA binding (in the absence of ethidium bromide), but this interaction could be abolished by performing the experiment in the presence of ethidium bromide. In contrast, the interaction between ATOH1 and E47 was stronger, more specific and could still be observed in the absence of DNA binding.

To test if GFI1 can enhance the transcriptional activity of ATOH1 independent of DNA binding, we used a luciferase reporter construct containing a multimerized AtEAM ATOH1-binding sequence (AtEAM) upstream from a minimal promoter. This multimerized sequence did not contain GFI1 binding motifs. We co-transfected the reporter construct with either *Atoh1* or *Gfi1* constructs in DAOY cells, a cerebellar medulloblastoma cell line, and performed luciferase reporter assays with either an *Atoh1* construct alone or co-expression of *Atoh1* and *Gfi1*. The reporter activity was significantly higher when *Atoh1* and *Gfi1* vectors were co-transfected compared to *Atoh1* alone (Fig. 6a). These results suggest that GFI1 can enhance the transcriptional activity of ATOH1 in vitro, but that GFI1 may be interacting with ATOH1 in an “off-DNA” manner, similar to the relationship observed between Atonal and Senseless in *Drosophila*^20^. Since GFI1 usually acts as a direct transcriptional repressor when bound to DNA, we next tested the effects of direct GFI1 binding to DNA on the transcriptional activity of ATOH1. We used a reporter construct with an enhancer containing both ATOH1 and GFI1 binding sequences upstream of a minimal promoter. We repeated the luciferase assay and found that reporter activity was significantly lower when ATOH1 and GFI1 proteins were co-expressed compared to ATOH1 alone (Fig. 6b).

We next sought to understand the nature of the interaction between ATOH1 and GFI1. We first performed a bimolecular fluorescence complementation assay (BiFC). We fused ATOH1 to the C-terminus of YFP, and GFI1 to the N-terminus of YFP, transfected the plasmids into DAOY cells and measured the fluorescence of the cells by flow cytometry. As a positive control, we also fused the N-terminus of YFP to E47, a bHLH protein that is known to directly heterodimerize with ATOH1. Transfection with ATOH1-C-YFP gave a very weak fluorescent signal, whereas co-transfection of ATOH1-C-YFP with E47-N-YFP produced a strong shift in the YFP signal (Fig. 6c). However, co-transfection of ATOH1-C-YFP with GFI1-N-YFP gave a significantly lower shift in YFP fluorescence intensity, suggesting that the interaction between GFI1 and ATOH1 may be weak and indirect. Non-specific interactions between nuclear proteins are known to occur in the presence of contaminating DNA, which can be eliminated by exposure to ethidium bromide^45^. We therefore tested the degree of direct physical association between GFI1 and ATOH1 in co-immunoprecipitation assays using ethidium bromide to prevent DNA binding. GFP-tagged ATOH1 was transfected into DAOY cells in the presence or absence of either Gfi1 or Flag-tagged E47 as a positive control. ATOH1-GFP was immunoprecipitated with anti-GFP camelid nanobodies, and the fractions probed with antibodies to either GFI1 or Flag to detect E47. The immunoprecipitation was performed in the presence or absence of ethidium bromide to inhibit non-specific DNA-dependent protein association. We found that GFI1 could be immunoprecipitated with ATOH1, but that this interaction was DNA-dependent and abolished by ethidium bromide (Fig. 6d). In contrast, the interaction between ATOH1 and E47 was not disrupted by ethidium bromide (Fig. 6d), suggesting that ATOH1 and E47 can interact strongly in the absence of DNA, but that ATOH1 and GFI1 interact weakly or in a DNA-dependent manner. This suggests that ATOH1 and GFI1 co-exist as part of a transcriptional complex that activates hair cell gene expression but do not bind each other directly.

## Discussion

GFI1 is an essential hair cell transcription factor that is induced by, and can cooperate with, ATOH1 to activate hair cell gene expression and differentiation both in vitro and in vivo^19,33,34,36,37,46^. This relationship between the two transcription factors is evolutionarily conserved, as a similar interaction has been observed between Senseless and Atonal^47^. However, unlike *Drosophila,* vertebrate GFI1 proteins have acquired an N-terminal SNAG repression domain and usually act as transcriptional repressors^29,48^. In this study we attempt to reconcile the dual genetic and biochemical functions of GFI1 as an activator and a repressor in the same cell type. GFI1 can co-operate with ATOH1 to activate hair cell genes without itself binding to DNA, a mechanism also seen in *Drosophila*^20^. In addition, it can repress non-hair cell genes^30^, as well as attenuating the expression of some genes expressed in nascent hair cells but which are down-regulated as hair cells mature; this activity involves GFI1 acting in its canonical role as a transcriptional repressor that directly binds DNA.

Several lines of evidence support the proposed dual roles and functional mechanisms of GFI1 in hair cell differentiation. First, our CUT&RUN data show that ATOH1 and GFI1 can bind to many of the same loci in hair cells. We believe that the number of co-bound peaks identified in our study may be an underestimate, as it has been challenging to find antibodies to GFI1 that work well in CUT&RUN or ChIP assays; this is demonstrated by the number of called GFI1 peaks we observed in our CUT&RUN experiments being five times lower than the number of peaks we observed with ATOH1/GFP CUT&RUN. Second, these GFI1/ATOH1 co-bound peaks show little evidence for GFI1 DNA binding motifs, suggesting that GFI1 is acting to promote ATOH1 function in an off-DNA manner. Third, we used two independent methods (bimolecular fluorescence complementation and co-immunoprecipitation) to suggest that the interaction between GFI1 and ATOH1 is significantly weaker than that of ATOH1 and its cognate E proteins, and likely occurs independently of GFI1 binding DNA. Finally, we show that GFI1 can indeed bind directly to DNA sequences close to hair cell gene loci, and that in some cases these genes are upregulated in *Gfi1* mutants, suggesting that here GFI1 is acting as a canonical repressor of hair cell genes. The degree to which these activating and repressing functions of GFI1 are both required for hair cell differentiation are intriguing—for example, a point mutation in the SNAG domain of GFI1 causes hair cell defects that are as severe as the *Gfi1* knockout mouse^46^, suggesting that the SNAG domain (and presumably its repressive function) is critically important for hair cell development. However, other domains of GFI1 are clearly also necessary for hair cell formation—for example, although replacement of the *Gfi1* coding region with that of its close relative, *Gfi1b*, causes hair cell defects, this replacement completely rescues other functions of *Gfi1*, such as its role in hematopoiesis^46^.

It is notable that at least some of the hair cell genes that can be repressed by GFI1 are only expressed transiently in developing hair cells and are downregulated as hair cells mature. Since *Gfi1* is a direct target of ATOH1^44,49^, this suggests that GFI1 can act in a negative feedback loop to attenuate expression of some early hair cell genes, but also in a feed-forward manner to enhance ATOH1 activation of other hair cell genes. Recently, POU4F3 was also shown to act in a feed-forward manner to promote hair gene expression with ATOH1^49^, but the nature of the feed-forward mechanisms exhibited by POU4F3 and GFI1 are very different: GFI1 is induced by ATOH1 and then forms part of a complex with ATOH1 and E proteins (this study), whereas POU4F3 is induced by ATOH1 but then acts as pioneer factor to render many ATOH1 targets transcriptionally accessible^49^. Since these three hair cell transcription factors can co-operate to promote hair cell formation more efficiently than ATOH1 alone or in pairwise combinations^19,33,36,37^, it is likely that both feed-forward mechanisms are operating together as hair cells differentiate.

By what mechanisms can GFI1 activate gene expression in an “off-DNA” fashion and repress gene expression by binding to DNA? The repressive mechanism of vertebrate GFI1 proteins has been well characterized^29,48^. In brief, the N-terminal SNAG domain of GFI1 has a similar structure to the tail region of histone H3, allowing it to preferentially interact with LSD1 histone demethylases^24,50^. Regions between the SNAG and zinc finger domains can also bind and recruit histone methyltransferases such as G9a^25,51^. It has been suggested recently that the catalytic function of LSD1 is not required for GFI1 to repress transcription, and that LSD1 may recruit histone deacetylases to GFI1 targets in the absence of demethylase activity^52^. GFI1 has been reported to activate gene expression in myeloid lineage cells in co-operation with C/EBP proteins^53^, but this interaction requires GFI1 to bind DNA directly. The mechanism by which GFI1 can enhance the action of ATOH1 without binding DNA is less clear. GFI1 can interact with the MIZ-1 transcription factor to repress cell cycle regulations such as CDKN1A and 2B^54,55^, and this interaction, which is believed to occur through GFI1’s C-terminal zinc fingers, does not require GFI1 to bind DNA^55^. GFI1 can also act with another zinc finger transcriptional repressor, PRDM5, through their respective zinc finger domains; this interaction can paradoxically lead to the activation of transcription^56^. *Drosophila* Senseless protein lacks a SNAG domain and requires its zinc fingers to interact with ATOH1^20^, although it is not known if this interaction is direct. Our data suggest that if GFI1 is indeed physically interacting with ATOH1, its interaction is far weaker than the interaction between ATOH1 and E47 through their helix-loop-helix domains. Alternatively, GFI1 could be interacting with another, currently unknown, component of the ATOH1-E47 transcriptional complex. Finally, it is possible that, as in *Drosophila*, the interaction between GFI1 and ATOH1 can enhance or modulate the specificity of ATOH1 for particular E-box DNA binding motifs^23^ or help recruit alternative E proteins to heterodimerize with ATOH1.

The divergence of vertebrate and invertebrate lineages was accompanied by the emergence of secondary mechanosensitive receptor cells such as hair cells and Merkel cells that can form synapses but have lost neuronal features such as axons and dendrites^5^. We speculate that the emergence of these secondary receptor cells may have been facilitated by the acquisition of a new repression domain in vertebrate *Gfi1* orthologues, which would have allowed GFI1 proteins to directly repress aspects of the neuronal differentiation program^30^ that were no longer required in secondary receptor cells. However, the association between GFI1 and ATOH1/E proteins in a positively acting transcriptional complex remained conserved in vertebrates, and allowed GFI1 to continue to promote the differentiation of genes required for mechanosensory cells.

## Materials and methods

### Animals

*Atoh1*^*A1GFP/A1GFP*^ (MGI: *Atoh1*^*tm4.1Hzo*^; Jackson Laboratories stock number 013593) mice were generated as previously described^41^ *Gfi1-Cre* (MGI: *Gfi1*^*tm1(cre)Gan*^) mice were generated as previously described^57^ and obtained from Dr. Noah Shroyer, Baylor College of Medicine, with the permission of Dr. Lin Gan, University of Rochester. Males and females were used between P0 and P1 unless stated otherwise. The Baylor College of Medicine Institutional Animal Care and Use Committee approved all animal experiments, which followed the recommendations of the American Veterinary Medical Association (AVMA) Guidelines on Euthanasia. The the study is reported in accordance with ARRIVE guidelines (https://arriveguidelines.org).

### Plasmid construction

#### Expression constructs

To express ATOH1 protein fused with GFP, the fusion gene was amplified from the pCDNA3.1-Atoh1-GFP^43^ and subcloned into the pEF1/V5 plasmid to create pEF-Atoh1-GFP using the In-Fusion cloning protocol. To make a Gfi1-2A-tdTomato expression construct, *Gfi1* was amplified from the pCMV-*Gfi1* vector (a gift of Dr. Melih Acar) with the addition of 20 bps of overlapping T2A sequence added to the beginning of the reverse primer. The tdTomato sequence amplified with the whole T2A sequence added to the forward primer, and subcloned into the pEF1/V5 plasmid to create pEF-*Gfi1*-T2A-tdTomato. TdTomato and GFP were separately subcloned to the pEF1/V5 plasmid to create control vectors. These plasmids were used for both the luciferase assays and the co-immunoprecipitation assays in Fig. 6.

*Reporter Constructs:* For the luciferase reporter construct containing only ATOH1 binding motifs, three multimerized AtEAM sequence^43^ were subcloned into the multiple cloning sites of the pNl3.1 NanoLuc construct (Promega) to create pNl3.1-3xAtEAM. For the reporter construct containing both ATOH1 and GFI1 binding motifs, an endogenous sequence containing both ATOH1 and GFI1 binding domains (5′-ACAGATGGTTGTGAGCCACTATGTGGTTGCTGGGATTTGA-3′) was subcloned into the pNL3.1 NanoLuc construct to create pNl3.1-AGmotifs.

### BiFC constructs

For bimolecular fluorescence complementation, *Atoh1*, *E47* and *Gfi1* were cloned into the BiFC vector as previously described^58^. Briefly, the *Atoh1* coding region was fused to the C-terminal portion of the YFP sequence (amino acids 175 to 239) to create Atoh1-cYFP. Both E47 and *Gfi1* coding regions were fused to the N-terminal portion of the YFP sequence (amino acids 1 to 174) to create E47-nYFP or Gfi1-nYFP. The complementation experiment was performed as previously described^58^.

### Luciferase assays

DAOY cells (ATCC) were transfected using Lipofectamine LTX and Plus Reagent (Invitrogen) using the manufacturer’s instructions with a small modification. Briefly, 50,000 cells were growing in a 24-well plate for 24 h. For the reporter assay, 25 ng of NanoLuc reporter, pNl3.1-AtEAM, and 25 ng of firefly reporter, pGL4.53, were transfected with 200 ng of pEF-Atoh1-GFP and 100 ng of pEF-Gfi1-tdTomato or matching concentration of control vector pEF-tdTomato. After 24 h, cells were lysed, and the luminous activity was measured using the NanoLuc luciferase reporter assay (Promega). Each experiment was repeated five to eight times and each measurement was performed in duplicates.

### Co-immunoprecipitation experiments

HEK-293 T cells were maintained in DMEM with 10% Fetal Bovine Serum (Thermo Fisher) containing 100 units/ml penicillin/streptomycin in a humidified incubator at 37 °C with 5% CO_2_. For immunoprecipitation assays, approximately 2.2 × 10^6^ cells were grown overnight in a 100 mm^2^ dish for each transfection. The cells were transfected with pCDNA3.1-Atoh1-GFP with pEF-tdTomato and pCDNA3.1-Flag constructs (control) or with various combinations of pEF-Gfi1-T2A-tdTomato and pCDNA3.1-E47-Flag (a gift of Huda Zoghbi) vectors using Lipofectamine LTX as per manufacturer’s instructions (Invitrogen). 48 h post-transfection, cells were washed once with PBS and lysed for 20 min in CoIP lysis buffer (25 mM Tris–HCl, pH 7.5, 150 mM NaCl, 1 mM EDTA, 1% NP-40, 5% Glycerol, 1 mM DTT and phosphatase inhibitors) on ice. Lysates were centrifuged for 15 min at 4 °C to collect the supernatant. GFP-Trap Magnetic Agarose beads (Chromotek, gtma-20) were equilibrated with CoIP lysis buffer by washing twice. 500 μg lysate was used for each immunoprecipitation, and each immunoprecipitation was performed in duplicate in the presence or absence of 100 μg/ml EtBr, with the volume made up to 500 μl with CoIP lysis buffer containing phosphatase inhibitors. 10 μl equilibrated GFP-Trap Magnetic Agarose beads was added to each tube, which was then incubated on an end-on rocker at 4 °C overnight. The tubes were placed in a magnetic separator and the captured beads were washed 3 times with wash buffer (10 mM Tris–HCl, pH 7.5, 150 mM NaCl, 0.5 mM EDTA). After the last wash, the beads were resuspended in 20 μl PBS and 5 μl of 6X SDS sample buffer. The resuspended beads were boiled for 5–8 min to elute the protein complexes. The beads were collected by placing tubes in the magnetic separator and the eluates were carefully transferred to the fresh tubes which were loaded on an SDS-PAGE gel for Western analysis. The following antibodies were used for western blotting: FLAG (Sigma F3165, 1:10,000), GFI1 (a gift of Dr. Hugo Bellen, 1:2000), GFP (Abcam 13970, 1:5,000) and GAPDH (Millipore AB2302, 1:10,000).

### Purification of hair cells by FACS

Inner ears were isolated from postnatal day 1 *Atoh1-GFP* animals^41^ under sterile conditions, and the cochleae were dissected in pre-chilled Ca^2+^- and Mg^2+^-free PBS (Thermo Fisher) under a dissection microscope. After the removal of the spiral ganglion and the lateral wall, 8–10 cochlear organs were pooled and digested with 200μL 0.125% Trypsin–EDTA (Invitrogen) at 37 °C for 8 min. Enzymatic digestion was terminated by the addition of 100μL 10% FBS (Invitrogen), and the digested organs were triturated with a P200 pipette to a single-cell suspension. After passing through a 40 μm cell strainer (VWR International), the single-cell suspension solution was loaded into BD FACSAria II to FACS purify GFP + hair cells into PBS supplemented with 10% FBS. The purity of the sorted cells was verified to be greater than 90% by re-sort and by cell counting under a fluorescent microscope.

### CUT&RUN library construction and sequencing

The CUT&RUN protocol for GFI1 binding across the whole genome was previously described (Skene and Henikoff, 2017). Briefly, FACS-purified cells (> 10,000 cells) were pelleted by spinning at 600×*g* for 5 min, washed twice with wash buffer (20 mM HEPES pH 7.5; 150 mM NaCl; 0.5 mM Spermidine), bound to Concanavalin-coated magnetic beads (Bangs Laboratories), permeabilized by 0.06% digitonin (Millipore) in the wash buffer, and then incubated with rabbit anti-GFI1 (Abcam, 1:100 dilution in wash buffer containing digitonin) at 4 °C overnight. After a brief wash, Protein A-MNase (homemade as described^59^) was added to cells at a final concentration of 700 ng/ml, followed by 1-h incubation at 4 °C on a Nutator. Cells were washed three times with wash buffer supplemented with digitonin, and then enzymatic chromatin digestion was activated by the addition of CaCl_2_ at a final concentration of 2 mM. The digestion process was carried out on the ice for 30 min and then stopped by adding the same volume of 2X STOP buffer (340 mM NaCl, 20 mM EDTA, 4 mM EGTA, 0.05% Digitonin, 100 μg/ml RNAse A (Thermo Fisher Scientific), 50 μg/ml Glycogen). Fragmented DNA was released from cells by incubation at 37 °C 300 rpm for 30 min on a thermomixer, extracted from the solution using the Phenol–Chloroform method, and dissolved in 40μL low-EDTA TE buffer (Swift Biosciences). Finally, extracted DNA was used for library construction with an Accel-NGS 2S DNA library kit supplemented with MID indexed adaptors (Swift Biosciences). The resulting GFI1 CUT&RUN library was sequenced on a NextSeq 500/550 platform (Illumina) for a total of 30 million 37 bp paired reads. Encode ChIPseq pipeline (https://github.com/ENCODE-DCC/chip-seq-pipeline) with the implementation of umi-tools^60^ was used for fastQC, read alignment to the mm10 mouse genome.

### Data analysis and visualization

Translatome data from Gfi1 null and heterozygote mice has been published previously^30^; GEO accession number GSE135760. For analysis of CUT&RUN data, BamCoverage was used to generate a coverage bigwig file from the BAM files to create custom tracks to visualize in the UCSC genome browser. The peaks were called by model-based analysis of ChIP-Seq (MACS2)^61^ with p = 0.01 cutoff and normalized with control IgG data. Peak annotation was performed using annotatePeaks.pl (Homer). The annotated compiled peak files were used to identify which genes containing accessible peaks. We then identified the unique and overlapping peaks between each group using bedtools. Motif enrichment analysis and individual condition peak calling were conducted with Homer. Heat maps were generated using online resources (http://www.heatmapper.ca/). The DAVID web tool^62^ was used to identify enriched GO terms. MEME suite 5.3.2 was used for ATOH1 and GFI1 motif analysis of individual peaks. Sequences of the peaks were extracted from the UCSC genome browser as input files. The ATOH1 binding motif (MA0461) and Gfi1binding motif (MA0038) were extracted from the JASPAR database as the motif input. FIMO (find individual motif occurrence) was used to identify the occurrence with ATOH1 and Gfi1 motifs on our input sequences, motif matches were selected if p-value < 1E−4.

### In Situ hybridization

Bisected heads of neonatal mouse pups were fixed in 4% paraformaldehyde in PBS overnight at 4 °C, cryoprotected in 30% sucrose in PBS at 4 °C, embedded in OCT compound (Sakura Finetek), and cryosectioned at 14 μm. Sections were fixed in 4% paraformaldehyde in PBS, pH 7.2 for 10 min at room temperature, followed by three 5-min washes in DEPC-treated PBS. The sections were treated with 1 µg/ml Proteinase K in DEPC-PBS for 5 min at room temperature, followed by three 5-min washes in DEPC-PBS and re-fixation in 4% paraformaldehyde in PBS, pH 7.2 for 10 min at room temperature. Sections were acetylated in 0.25% acetic anhydride in 0.1 M triethanolamine, pH 8.0 for 10 min at room temperature, followed by three 5-min washes in DEPC-PBS. Slides were incubated in hybridization buffer (50% formamide, 5xSSC, 50 μg/ml Yeast tRNA, 100 µg/ml Heparin, 1X Denhardt’s Solution, 0.1% Tween 20, 0.1% CHAPS, 5 mM EDTA) for 1–2 h at 65 °C. 100 μl of digoxygenin-labeled Rasd2 probe (1 mg/ml) was added to each slide and the slides covered with glass coverslips. The slides were incubated in a chamber humidified with 5xSSC, 50% formamide at 65 °C overnight. Coverslips were removed by rinsing in 0.2xSSC and the slides washed in 0.2xSSC at 65 °C for 1 h. The slides were then washed in 0.2xSSC for 5 min at room temperature, followed by another 5-min wash in 0.1% Tween-20 in PBS (PTw). The slides were blocked in 10% lamb serum in PTw at room temperature for 1 h and then stained with anti-digoxygenin-alkaline phosphatase antibody (1:2000) for 1–3 h at room temperature in a humidified chamber. The slides were then washed three times for 5 min each in PTw and equilibrated with freshly-made alkaline phosphatase buffer (100 mM Tris pH 9.5, 50 mM MgCl_2_, 100 mM NaCl, 0.1% Tween 20) for 10 min. The slides were developed in alkaline phosphatase buffer containing 0.33 mg/ml NBT and 0.18 mg/ml BCIP in the dark at room temperature until the purple reaction product had developed to a satisfactory degree. The reaction was stopped by washing the slides in PBS three times for 15 min each, followed by fixation in 4% paraformaldehyde in PBS, pH 7.2 for 30 min. The slides were then rinsed and mounted in 80% glycerol in PBS.

### Chromatin immunoprecipitation and Q-PCR

Chromatin immunoprecipitation was performed using the “microChIP” protocol of Dahl and Collas^63^. Cross-linked sensory epithelia were lysed in 120uL lysis buffer (50 mM Tris–HCl, pH 8.0, 10 mM EDTA, 1% SDS with fresh 1% protease inhibitor and 1 mM PMSF) and incubated for 15 min on ice. The samples were sonicated using a Bioruptor (Diagenode) programmed for 30 s on and 30 s off, for 15 cycles, with vortexing after every 5 cycles. Four-hundred microliters of RIPA ChIP buffer (10 mM Tris–HCl, pH 7.5, 140 mM NaCl, 1 mM EDTA, 0.5 mM EGTA, 1% Triton X-100, 0.1% SDS, 0.1% Sodium deoxycholate, 1% protease inhibitor and 1 mM PMSF) was added to the tube. Samples were centrifuged at 12,000* g* for 10 min at 4 °C. The supernatant was collected into a new tube and the pellet was re-extracted with another 400uL of RIPA ChIP buffer. To precipitate Atoh1-GFP bound DNA, 10uL of GFP magnetic beads (Chromotek, GFP-Trap Magnetic Agarose #gtma20) were washed twice with RIPA ChIP buffer (10 mM Tris–HCl, pH 7.5, 140 mM NaCl, 1 mM EDTA, 0.5 mM EGTA, 1% Triton X-100, 0.1% SDS, 0.1% Sodium deoxycholate, 1% protease inhibitor and 1 mM PMSF). To precipitate Gfi1 bound DNA, 10uL of Magna ChIP Protein A + G Magnetic beads (Millipore#16–663) were washed twice with RIPA ChIP buffer (10 mM Tris–HCl, pH 7.5, 140 mM NaCl, 1 mM EDTA, 0.5 mM EGTA, 1% Triton X-100, 0.1% SDS, 0.1% Sodium deoxycholate, 1% protease inhibitor and 1 mM PMSF). 200uL of chromatin sample was incubated with 10uL washed GFP magnetic beads (for Atoh1-GFP) and Magna ChIP Protein A + G Magnetic beads (with 2ug of IgG (Millipore) or Gfi1 (Abcam) antibodies) for overnight at 4 °C on a Nutator. After incubation, the beads were captured on a magnetic rack and the supernatant was removed. The beads were washed three times with 200uL ice-cold RIPA ChIP buffer at 4 °C on a nutator for 5 min each. After the final wash, 200uL TE buffer was added to the beads and incubated for 5 min at 4 °C on a nutator. Beads were captured on a magnetic rack. After removal of TE, 150 µl complete elution buffer (20 mM Tris–HCl, pH 7.5, 5 mM EDTA, 50 mM NaCl, 20 mM Na-butyrate, 1% (wt/vol) SDS, 50 µg/mL proteinase K. Na-butyrate, SDS, and proteinase K should be added just before use) to each tube and incubated for 2 h on a thermomixer at 68 °C, 1300 rpm. The beads were captured on a magnetic rack and the supernatant was transferred in a clean 1.5 ml tube. A second elution was done adding 150 µL complete elution buffer to the remaining beads and incubated on a Thermomixer for 5 min at 68 °C, 1300 rpm. The beads were captured again using a magnetic rack and the supernatant was combined with the first supernatant. 200µL elution buffer to the eluted ChIP material making it to 500uL.The DNA was extracted with an equal volume of phenol:chloroform:isoamyl alcohol. The final pellet was dissolved in 60uL TE. The sample was then stored at − 20 °C for up to 1 week or used directly for ChIP-qPCR with primers for the following genes: Rasd2 (Forward: 5′-TCCTGCTGGATCTTCACACC-3′; Reverse: 5′-GGTGGCACATGTCCTCAGAT-3′), Anxa4 (Forward: 5′-CTTTTACCTGCCCCGCCCA-3′; Reverse: 5′-GAAACGGCACCTGACCTGTTA-3′) and Atoh1 (Forward: 5′-CCAAGAAGCGTGGGGGTAG-3′; Reverse: 5′-GCTTCTGTAAACTCTGCCGG-3′). The fold enrichment for Atoh1-GFP or Gfi1 was calculated relative to the negative (IgG) sample, in other words the signal over background.

## Supplementary Information

Supplementary Figures.

Supplementary Table S1.

Supplementary Table S2.

Supplementary Table S3.

Supplementary Table S4.
