# Supplementary material for: GFI1 regulates hair cell differentiation by acting as an off-DNA transcriptional co-activator of ATOH1, and a DNA-binding repressor
Source: Sci Rep. 2022 May 12;12:7793. doi: 10.1038/s41598-022-11931-0 (PMC9098437; doi:10.1038/s41598-022-11931-0)
Supplement: Supplementary file 1 — Supplementary Figures. [file 41598_2022_11931_MOESM1_ESM.pdf]

Jen et al., Supplementary Figures:

Supplementary Figure 1: Gene ontology analysis of 672 non-hair cell genes that are up-regulated in hair cells in *Gfi1* knockout mice

Supplementary Figure 2: Known motif analysis of 732 *Gfi1* binding loci in hair cells.

Supplementary Figure 3: DNA sequences corresponding to the Rasd2 peaks in Figure 5 that contain binding sites for ATOH1 but not GFI1 transcription factors.

Supplementary Figure 4: ChIP-QPCR analysis of ATOH1 and GFI1 binding to the same distal regulatory elements in the *Anxa4* and *Atoh1* genes, both of which are direct targets of ATOH1. Cochlear epithelium from neonatal *Atoh1-GFP* mice were processed for ChIP using antibodies for GFP (to pull down ATOH1-bound DNA) and GFI1, with IgG as a negative control. QPCR amplification of the DNA was carried out with previously published primers to regions of the *Atoh1* and *Anxa4* loci [38]

Supplementary Figure 5: Full images of the blots of the co-immunoprecipitation experiments in Figure 6D. The labels are the same as those in Figure 6D

Jen et al., Supplementary Tables:

Supplementary Table S1: Sheet 1 – list of 1378 hair cell enriched genes (Figure 1A). Sheet 2: 90 hair cell-enriched genes up-regulated in the GFI1 knockout cochlea and 473 hair cell-enriched genes down-regulated in the Gfi1 knockout cochlea (Figure 1B)

Supplementary Table S2: Sheet 1 - List of 732 GFI1 binding loci with their closest mapped gene name. Sheet 2 - list of 3792 ATOH1 binding loci with their closest mapped gene name

Supplementary Table S3: Sheet 1 - List of hair cell loci with GFI1 binding sites that are either up-regulated (8 genes) or down-regulated (51 genes) in the Gfi1 knockout. Sheet 2 - List of hair cell loci with GFI1 and ATOH1 binding sites that are either up-regulated (4 genes) or down-regulated (40 genes) in the *Gfi1* knockout. Sheet 3 - List of non-hair cell gene loci with GFI1 binding sites that are upregulated (39 genes) in the *Gfi1* knockout

Supplementary Table S4: Sheet 1 - List of 350 loci uniquely binding GFI1 in hair cells. Sheet 2 - List of 386 loci binding GFI1 and ATOH1 in hair cells. Sheet3 - List of 3402 loci uniquely binding ATOH1 in hair cells.

GO analysis for 672 non-hair-cell genes upregulated in the Gfi1 knockout

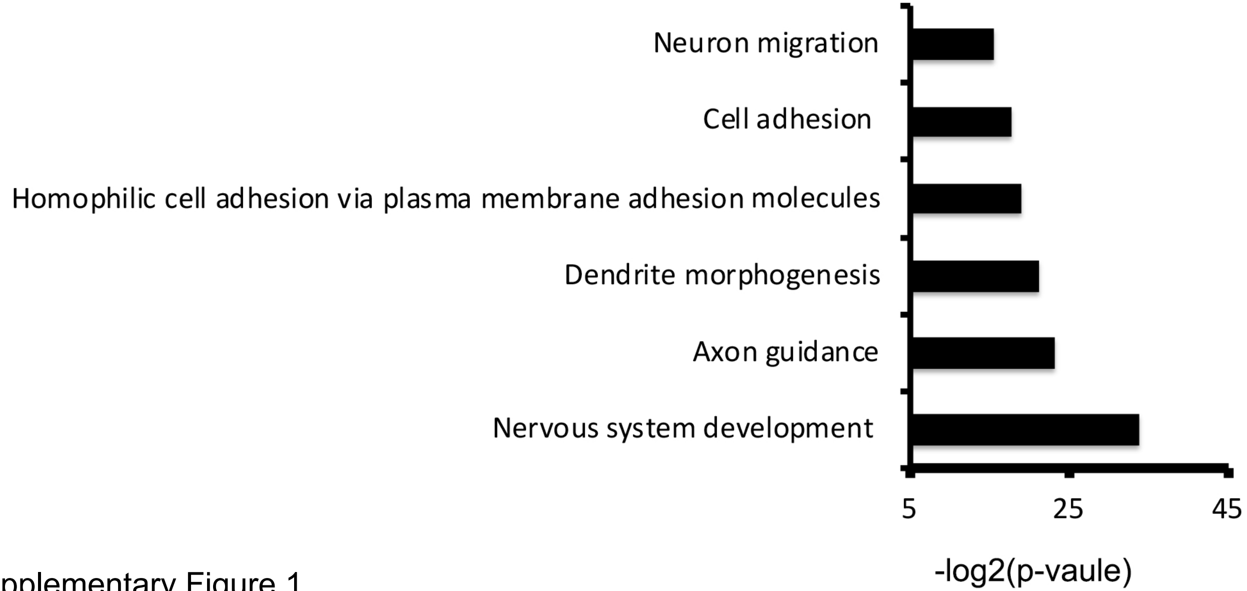

Supplementary Figure 1

Known motif enrichment analysis for Gfi1 binding loci

bHLH transcription factors	Motif	P-value
NeuroG2	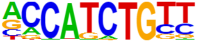	1e-12
NeuroD1	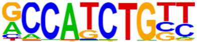	1e-11
Atoh1	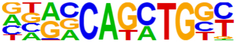	1e-11
Ascl1	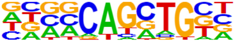	1e-10
BHLH15	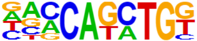	1e-10

Supplementary Figure 2

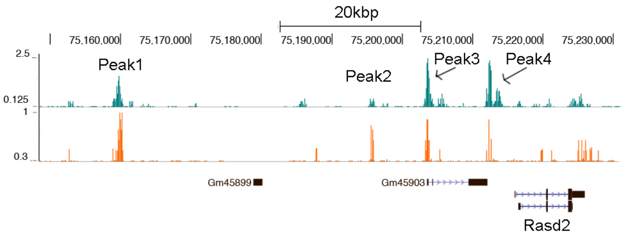

Peak 1(chr8:75157573-75159079)

```
CAAATCAGTTGCCCATCTCACTAAGTGCCTGTGCCTCTCCCTGGGTTGCT
GCTGAGCTGAGCATGGCAGGTAGAGTGTATGGCTATATGGGCTAGGGAAC
TCCAGGGGAACTCCGTGGGGACATTCCCGAAGCTCCCTGGAAGCTGCTAA
TTGAATCCTATTCTCTGCTCCCTGGGAGGCTGACAGCTGAAAGGAGCTCA
GCACAGAGATAATGAACTTGAGACCTCTGAAGTTGCTGCTCTCTTTGCAT
ACAAAGATCCTGGCCTCTATCCTGCACTTACTAAAAACTCCTGAGAGCTG
GGGCCAGGCAGCTTTGCCCAAAGGCGATCTGCCTGCTCTGTTCTAGGTCC
AGAAGGGCAATGATCTTGGGGAACTGTGCTCATCAATGCATGTCTCCTAG
GAGCTGCCCCTGCTGGCACCCAGCACCCACTCAGCATCCCACTCGTACAC
GGTGACGTGCTGGGTGGAGGGGGCAGAGAGGAGATGTCAACATGACTGAC
ATGCCAAATGCCATGTGAGCCTTGGAGTGCCACTCATCTGGGGGACTGAA
GAGCAAGCTGGAGCTACTATCCTGCCAGAGGGGACAGGTACAGCAGCTTC
TGTCCTGAAAGATCCTTGAACTCCCTGCCTCAATTTCTAAAGAGAAAGTG
AGGCTCAGAAAGACAAGATCAGTGTGCAGTGCCCAGGTTGGGGACACATA
TCAACTCTACAATTCATTGTTATGTGATTGCGTGAAGATAGGCCTATAAT
CCCAGTACAGGAGTGGAGGCAAAAGAATAAGGAACAGAATGTCATCCTTC
ACAGCATAGCAAGCTCCTAGCCAGCCTGGGCAACAGGTAAACTGTCCAAA
ACAAAGGAAACACTTGCCTAACAGCTTGAAAGCCTGGCTCCATCCTTATC
ACAGAATCAATCAGTCAAGTCTTATCATGCCTCTGGATAATAGACTCCTC
ATATACATGAGTAAATGGGCTCAGAGTGATTAAGGAAGATATCTTAGGTT
TGCACAGCTGGTGAATGGCAGAGCTAGACAGAGTTACATCTTTCACTGAC
CCTATATTAGTTGAGTCCAGCTACCTAGGGCCCCAGGCCCTCTGCGTCTC
CCTCGAGTCTAAGACTATGGGTGGGCTGTTATGTCTGCTTGGCATTCAGA
TGGGTTCTGATCCCCTGGCTCCTGTCACATGAACTTTACCCACTGAGCCA
TCTCACCAGCGTCTCATGGCAGAATTTCATGGTGGGTTGTTATTGTTGGA
CAGAAACTTGATTTTTAGGCTGTGTGTCTCAGGAACTCATCGCCCTTTTG
GAACTTGTGCTCTCTGGTTCCCATGGAGATGAGGAGAGCTGGTCCTTGCC
CAGCATGATACTAGGTTGGTTTGTTCTATTTGTAATCACAGAAGACTGTG
AGGTAAGGCCCTGGCCCTTGGGGACCTGGGGATGGGACCCTTAGCATGCT
CTTGGTGCACTCCAGCCTGAGATATCCCCATCAGAGAAGGGTGGGAGGGA
ATAAGTT
```

Motif ID	Alt ID	Sequence Name	Strand	Start	End	p-value	q-value	Matched Sequence
MA0461.1	Atoh1	mm10_dna	+	1005	1012	4.87e-05	0.141	CAGCTGGT

Peak 3 (chr8:75202167-75203672)

```
ACATGCAGTCAGATTCTTTTACTCGGAAGTGGGTTGCCTTCTAAAGGTCA
TCTCATGAAAGAAGTTGCTGGATCAGTGCAATGCCACGTCAACTTGATTG
CTGAGTCGACACACAGAACCTGGCAGGATAGCACATCGGAGGAGGTGGTT
ATTCACACTTGCTAGGTGACAGGCCACTTGTCTTTTATGGGTTCAATAAA
GATTGCTTTCAAGGTCTAAGAACACCATCATAGGACGATGGGCTGGCTGG
AGGGTTTCATGGAGATGAGAGGACGGGGAGGGGTTTGTAGATCATTAGTG
TTTGCTTTTTGAGGCAGGGTTTCATACAGCCCAGGGAGGCCTCAAGCTTG
ATATATAGCTGAGGTTATCCTTGATCCTCTGATCCTCCTGCCTCCACCTC
CCAAGTTCTGAAATTACAGATGTGTGGCACTATGCTTAAGCTATGTGCTG
CTGGGGATGGCACTCAGCATGCTAGGCAGGCACTACCAACTGAGCCATGC
CACCAAACCTTGTTTGTTTTTTCTTTCATTTGGGACCAGAGTTCACCTTG
TTATAGGTCTGGGCTATACAATAGCCATAGAGACAGATTTTGGTTGGTGT
GGGAGTGAGTACTGTCCTCCTGAGGTAGGGTGACAGAGTAGAGAGTGGAG
GATGGATACTGTGTCAGGGACCAATAGTCTTCATATAGACCTGTAGAAGC
TGGCTCCTACTTGGGAAATGTAAGCCTGACCAGTTATAAGGCTTGCCCCT
GCTTACCATCTGTTGAAGAAGGGTCACAGACATGCCACCTGGCTTCCTGG
GGGGAGATAGCATCTGCAAGACAGGATAGCTCTTCTGGGAGATGCTTGGC
TGGACTCAGGCATAATTTATTCAACAGCCTTAGACACCTGTACTCCGGGG
CCGGTGCTGGCCTTGGCTATTAGCCAGAACCCAGTTCTGGATACATGCCT
GTCTGTTTATCTGACCTGGGGATGTGAGATGTGGGGCAAAGGCCATCCAG
AGAAAGCACCTTACCCTACCTTGAACTGCTTGGATGAAGGTTGGAAGGGG
GAGTTTGTTTTGAGCCCTAGGTAGACCAGTATGGCCCGGTATTCACTGTC
TGCTGCAGAGGAGACAGTAGAAGACCTGGTGGCTCTGAATCCCAGAACCT
GAACAACCCCAGTATATCCTCTCTGCAAGCCTTCTAGTGGTCTCAGGACA
GACTCATGCAGCCAGGCTCTTGGCATAGCCATTCCTGCTGGATCTTCACA
CCTATAGAATGGAGAAGACCACCACATTCCATCAGTCTAAATCAGGATCA
GATCCTCCAAGTAACATGCCTGAAAAACACAGATGGCATCTGAGGACATG
TGCCACCTCCTCAACTGCTCACACTCATATTTTCAGCATTGTCACCCTGA
TCCTCAAATGTGGGGACATGGTCATGAGGTGGCCCAGAGGAGGGTTTCCT
GCCTTGAGGACTGAGCAAGGAGAATGACGCCATTCCATTTCCTCCTGTGG
CTACGA
```

Motif ID	Alt ID	Sequence Name	Strand	Start	End	p-value	q-value	Matched Sequence
MA0461.1	Atoh1	mm10_dna	+	1330	1337	2.2e-05	0.0634	CAGATGGC
MA0461.1	Atoh1	mm10_dna	-	755	762	4.87e-05	0.0703	CAGATGGT
MA0461.2	Atoh1	mm10_dna	-	755	764	5.71e-05	0.169	AACAGATGGT

Peak 2 (chr8:75194608-75195109)

```
TGTTTTGTGCTCTATGTCTCTGCTGTCACAATGCACAAAGGCATGTTGGA
CAATAGCTCTCCAGGGGATATAGCTCATCTCAGCCCCTCTATCAGGCAGG
CACAGATCTGTGAGCTCAGCCCTCCATCCTGGCTGCCTTCCACTACCCCA
CCTCTCCACCCTCCCCTGCCCCCCCCCCCCCCCCAGGCTCCTCTCTTCTG
AGGATGACTCACACCCTGCTGACTCCACTCAGCCCATCAACTGCTCTTCT
CTGCAACTGGAGGAGCTGCTGTCTCGCTTATTTATTGAAACTGGCAAACA
CAGGCTCTGAGTTAATAGGTACAAGAGGGTAGCCAGAGGAAAACCCCCTT
CCCACCTCCTTCCCCAGCCTGGCTCTGGGAGAGGATGATCCTTTGCATAT
GCTAATATGAAGCTGTCTAGACGCTTTGCTTTCCAGGTGCTGTGCACAAA
TGGTAGCCCTTAGAGCTAACAAACACCTGACACAGGTGACTTATTTATAA
```

Motif ID	Alt ID	Sequence Name	Strand	Start	End	p-value	q-value	Matched Sequence
MA1467.1	ATOH1(var.2)	mm10_dna	+	264	273	5.81e-05	0.0539	AGCTGCTGTC

Peak 4 (chr8:75211114-75212130)

```
TATATCACTGGGAAGCCTGCTCTTTTCTCAAGGGAAATGGAGGAACAGTA
GATTGGGGGGGGGGGAAGAGGAGTGATGCGGAGGGGGGTAGAGAGGTTT
GGTGGGAGGTGAGACTGTATTTGGAATGTATTGTATGAGAGAAGAATAAA
TAAATTTAAAGTGTGTATGTGTTAAAAAGAAGTTTTAGGTTCAGGGAAGA
GAGGGAGCCTTAGGGAGTACAAAGGAGGAGGAGGCTTCAACACGTGGTCC
ATCCGGAGAGACCTGAGTGTAGGGAAGGGGACGAACTTGGGTACATGTTG
GTGGGCTCCATGAAGTGTTCCTGGATGTGGAAGATTTCTGTGCACCTGCC
TGGTAGCTGAGCTAATTTTTTTTTTTTTTTTTAAACCAGGCTGAAGCAGG
GTCAGGTGGGTCCCCAGGGCCAGGCAACAACATGTGCATTGCCAGCCCAA
TGTGGATCTGGATCTGGTCACTGGGGTGTGACCCACTCATCGAGGAGGAC
TTGGCTGAAAGCCCAAGGATTGGGTTCCTGTGCCAGCTCAGAGCTGGCCA
CCTGCCACACTCCCAAGTGGAGGGTGGAAAGTTGCTTGGTGGCCTGAGTC
CTCCCAGCTGCAGCCTTCCAGCTGAGGCACCCTGCTCTGGCTGCCTCCTG
CTGCCAAGCTCACAGGCTCTGTGCGTCAGGCCCAGTGCAAACTCTCCGGG
CTAAAGCTAGTTCCTAGAAGTCAAGTCTGGTTTGGGAAAGGGCACAGGAC
TAGGATTTTGCCATTTGTTTGCTACTGGTGACCTTGGGCAAGTCTCTTAG
CTACATTGAAGCCTAGACTTAGGCCTTAAACCCCGGCCCAAGGGGACCGG
CAAACCACCTCTTCCTGGCCCCTCCCCCATCCCGATGCATCTTCTCTCAT
ATAGTATTCCTGGAGGATGTCAAGATACTTTAATGTTTGATGAGCAACCC
ATTTCCTGTGTTTTAAGCCCAAGGCCATGTTAACGATTTTGTTACTCAAT
GGACTTGGCAGTATTGA
```

Supplementary Figure 3

Supplementary Figure 4

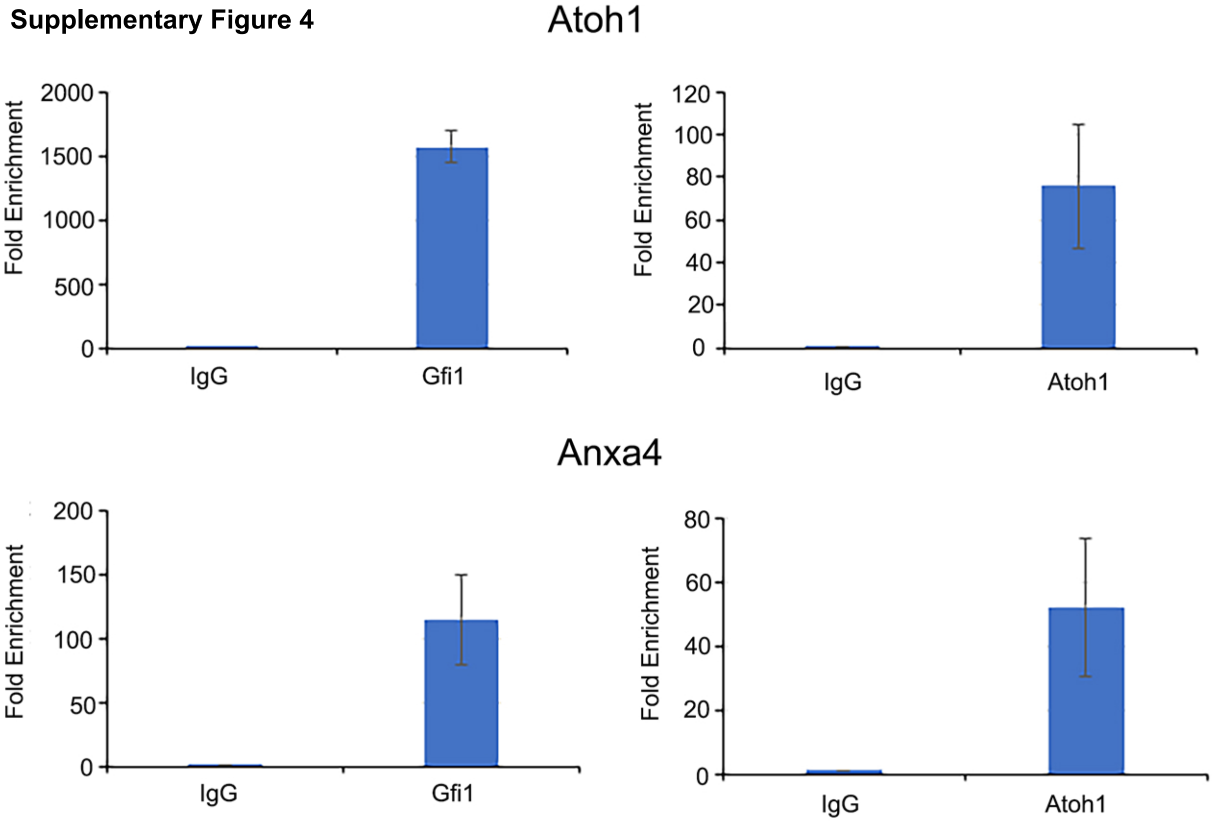

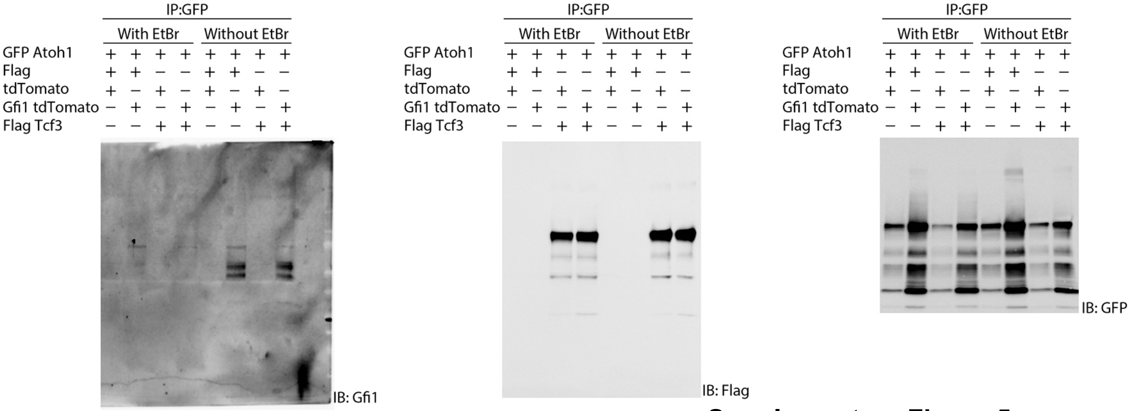

Supplementary Figure 5
